# Assessment of Occupational Health Hazards Due to Particulate Matter Originated from Spices

**DOI:** 10.3390/ijerph16091519

**Published:** 2019-04-29

**Authors:** Era Upadhyay, Afnan Ahmad Mohammad AlMass, Nandita Dasgupta, Safikur Rahman, Jihoe Kim, Manali Datta

**Affiliations:** 1 Amity institute of Biotechnology, Amity University Rajasthan, Jaipur, Rajasthan 302 002, India; eupadhyay@jpr.amity.edu; 2 Emergency Medicine Department, King Saud University Medical City, King Saud University, Riyadh 11321, Saudi Arabia; afnan.almass@hotmail.com; 3 Department of Biotechnology, Institute of Engineering and Technology, Dr. APJ Abdul Kalam Technical University, Lucknow, Uttar Pradesh 226031, India; nanditadg254@gmail.com; 4 Department of Medical Biotechnology, Yeungnam University, Gyeongsan 712-749, Korea

**Keywords:** spices, spice allergens, spice industry, occupation, respiratory health

## Abstract

Spices have been known for their various health activities; however, they also possess the allergic potential for the respiratory system and the skin as they are fine particulate matter. Persons involved in spice agriculture and food industries are at greater risk since they are exposed to a considerable amount of combustible dust, which may be the cause of fire and explosion and adversely affect the health. These workers may experience allergy, long-term and short-term respiratory issues including occupational asthma, dermatitis, etc. Some spices induce T cell-based inflammatory reaction upon contact recognition of the antigen. Antigen Presenting Cells (APC) on binding to the causative metabolite results in activation of macrophages by allergen cytokine interleukin (IL)-12 and tumor necrosis factor-beta (TNF). Cross-reactivity for protein allergens is another factor which seems to be a significant trigger for the stimulation of allergic reactions. Thus, it was imperative to perform a systematic review along with bioinformatics based representation of some evident allergens has been done to identify the overall conservation of epitopes. In the present manuscript, we have covered a multifold approach, i.e., to categorize the spice particles based on a clear understanding about nature, origin, mechanisms; to assess metabolic reactions of the particles after exposure as well as knowledge on the conditions of exposure along with associated potential health effects. Another aim of this study is to provide some suggestions to prevent and to control the exposure up to some extent.

## 1. Introduction

Spices are dried and processed variants of barks, roots, seeds, and fruits of the different crops. Initially, small-scale cultivation sufficed the demand of the population, but with the world-wide acceptability and demand, large scale plantations and associated processing units have developed. Currently, India is the largest producer, consumer, and exporter of spices and spice products [1]. The International Organization for Standardization (ISO) have recognized 109 spices in the world, out of which, more than 55 spice crops are grown in India, thus contributing to 20%–25% of the world trade in spices [2,3]. The raw spices undergo pulverization, grinding and sifting procedures to obtain powdered consistency. These procedures lead to the release of suspended particles along with volatiles, thus creating a niche for distinct chances of occupational exposure in processing industries. There has been a progressive increase in allergy patients from 1964 (10%) to 2011 (20%) and is expected to rise to 50% by 2050 [2,3].

Spices are generally added to enhance the flavor and taste of food. However, they also contain some irritants and pharmacologically active ingredients that can activate the immune response [4,5]. In general, the tiny particles are considered as dust but due to different nature, origin, source, mechanism of generation, and uses of spices, we have tried to determine the specific term for the particles released from spices during processing and seasoning. Usually, the size of dust particles ranges from about 1 to 100 µm in diameter, and they settle slowly under the influence of gravity. WHO does not categorize spice as dust; on the contrary, other organizations like OSHA, ISO 4225-ISO and IUPAC recognize airborne spices as dust. As ground spices encompass all properties in compliance with definitions, it may safely be addressed as a component of anthropogenic aerosol.

**Figure 1:**
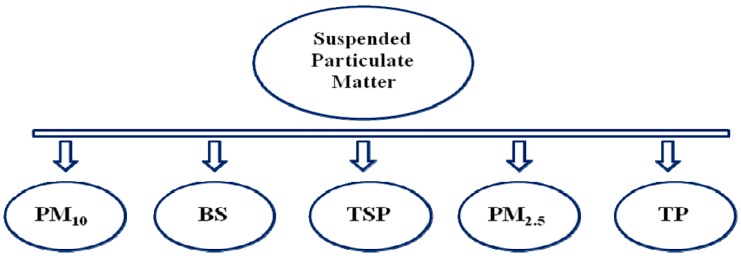
Categorization of Combustible Dust based on origin, processing and uses of spices (TSP—total suspended particles, PM_10_—particulate matter size less than 10 µm, PM_2.5_—particulate matter size less than 2.5 µm, BS—black smoke, TP—thoracic particle).

Spice particles vary in the range of 75–1000 µm depending on their processing methods [6,7,8]. Based on origin, processing, and particulate size, spices may be categorized into five classes-Total Suspended Particles, PM_10_, PM_2.5_, black smoke and thoracic particles (Figure 1). This classification complies with international agreement among the American Conference of Governmental Industrial Hygienists (ACGIH), the International Organization for Standardization (ISO), and the European Standards Organization (CEN) and thus correlates the inhalable, thoracic and respirable fractions [9,10,11,12].

An essential parameter decisive of seasoning potential of spices is its ‘flowability’ property. This plays a significant role during handling and processing operations as flowable powders tend to disperse with altered rates depending on the time and limit of exposure to air during processing. As it is an important determinant, theoretical simulations and experimental studies have been performed for evaluation of different bulk solids flow properties testers. Various mathematical equations have been cited to determine the particle size distribution of powder like Rosin-Rammler-Bennett, Gaudin-Schuhmann, Gaudin-Meloy to name a few [13]. Flowability is directly correlated to the aerodynamic diameter, thus plays an equally important role in dust exposure in its airborne state [14]. Additionally, dust particles tend to act as a conduit for the transmission of allergenic proteins [15] and as an adjuvant for immuno-stimulatory responses. Hence the spatial and temporal variability of these suspensions decides the intensity of allergenic responses. Symptomatic allergic reactions have been revealed through dermatological, gastrointestinal or neurological symptoms due to occupational exposure to spice dust [16,17].

Thus, with increasing risks and incidence, it becomes imperative to understand the transmission and molecular mechanism leading to allergic manifestations due to spices. Furthermore, the review highlights the impact and assessment of control strategies in curbing the adverse effect of occupational spice allergens.

## 2. Exposure Assessment

**Figure 2:**
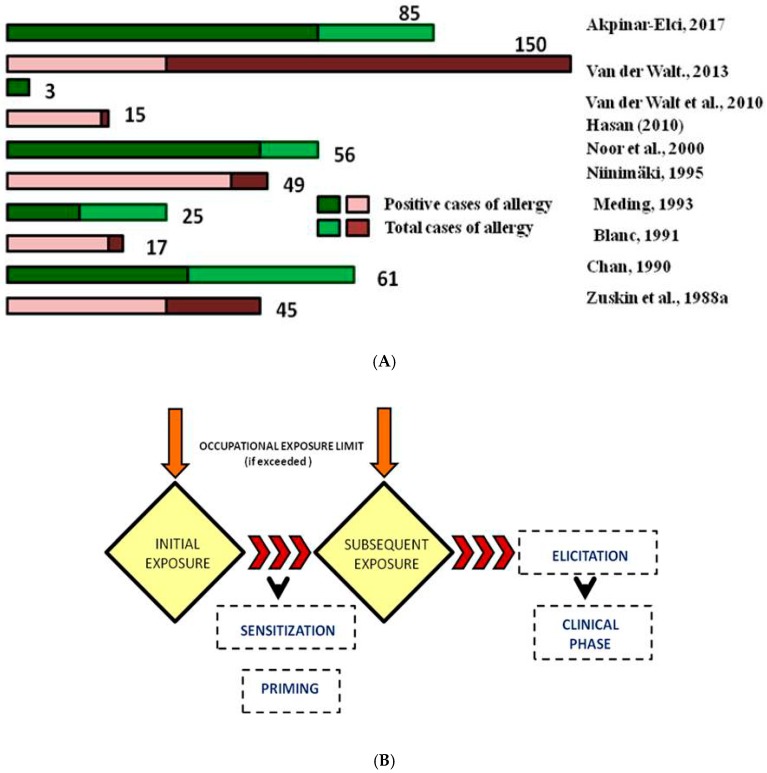
(**A**) Semi-quantitative analysis of occupational exposure to different spices from different sites. (**B**) Process flow of symptomatic allergic response from initial sensitization.

Spices are a particulate matter that is associated with various respiratory problems and lung infection [18,19]. The exposure was assessed by the prevalence of incidence rate of all occupations in the spice processing and packaging industries. Inhalation and/or dermal contact of suspended particulate matter from spice fields and spice processing were found to be the nodal cause for the initiation of allergic symptoms. Additionally, intake of spices as a part of a ‘generally recognized as safe’ [GRAS] additive has progressively heightened (Andreozzi et al., 2019). With inhalation, the concentration of the allergen from the site of emission varies to the site of exposure, which may be measured semi-quantitatively as low, medium, or high levels. Coherent calculation of allergen distribution in the atmosphere is calculated statistically, allowing the possibility of non-homogeneity in distribution (Figure 2A). Exposure assessment to aeroallergens has been experimentally monitored by enzyme immunoassays (EIA) and halogen immunoassay (HIA) in specialized laboratories. Vacuum sampling with dust cassettes enables concentrated dust sampling for EIA and HIA based analysis [20]. HIA involves the visualization of carrier particles with the allergens by immunostaining with human IgE [21]. The value is expressed as micrograms of allergen per gram of dust (µg/g) or as an area concentration in micrograms of allergen per m^2^ (µg/m^2^). Thus, sampling enables documentation (Figure 2A), giving precise ranges for a type of allergen and its corresponding concentration required to elicit a response (Figure 2B).

The usual procedure of inhalation exposure assessment involves correlating the concentration of the SPM with the incidence and persistence of allergic reactions. Various spirometry results like FVC, FEV1, FEV1/FVC ratio, FEF25–75, PEFR, FIVC, and PIFR assess the lung function and enables with allergen exposure [22]. As per the seasoning and spice association [23], the testing matrix for spices and herbs is complex because of its propensity for cross-reactivity. Fractional exhaled nitric oxide (FeNO) is a prognostic mechanism for analyzing lung functioning. In a study of 150 samples, 13% were monosensitized, and 6% were co-sensitized to garlic and chili pepper. A marked baseline preshift FeNO was having the geometric mean, 14.9 ppb. FeNO tends to increase 24 h after exposure to the allergen and measurement of FeNO is recognized as an accurate, reproducible, and important non-invasive surrogate marker for airway inflammation. There was almost 12% increase within 24 h thus demonstrating a strong association with elevated levels of suspended spice dust [24]. Similarly, in a study conducted in Grenada, occupational exposure and respiratory health problems among nutmeg production workers were observed in Grenada. Approximately half of the workers were found with work-related lower respiratory symptoms such as dry cough (49.4%) and shortness of breath (42.9%). High prevalence of respiratory symptoms among workers in the existing facility was consistent with measured levels of dust and mold and, was widespread overall work areas [25].

## 3. Spice Allergens

Spices contain a wide variety of substances with potential allergenicity, causing a variety of reaction categories as non-immunologic or immunologic responses [26]. The non-immunologic response includes irritation effects on the skin. Immunologic response after ingestion of spices induces IgE mediated (rhinoconjunctivitis, asthma, urticarial, angioedema, anaphylaxis, etc.) responses similar to food allergy [27,28,29]. Over the years, class I and Class II allergens were discovered from various sources [30] and in spice, they have been categorized into phytochemicals and proteins. Inflammatory pathway associated with phytochemicals is induced with the release of NF-KB [31]. Phytoconstituent of chili, capsaicin, was implicated as one of occupational allergen first time in 2012 [32]. The vanilloids present in hot spices (black pepper, paprika, cayenne, and chili) tend to aggravate the allergic symptoms by compromising the protective barrier function of the mucosa. In the presence of an allergen, Scoville index of the chillies decides the sensitization potential for the allergens with molecular weight less than 70 kDa. Its adjuvant potential enables the easier absorption of the allergen across the mucosal membrane, thus inducing epitope sensitization [5,33]. However, earlier studies on the physiological effects of capsaicin have been observed to cause a reduction in conductance via specific airways [34]. Broncho-constriction by capsaicin for an extended period resulted in neurogenic inflammation and eventually to non-IgE based rhinitis [35].

Cinnamic aldehyde, an aromatic compound present in cinnamon and curcumin, a lipophilic polyphenol from turmeric induces T cell-based inflammatory reaction upon contact recognition of the antigen. The causative metabolite, which when processed by antigen presenting cells (APC) in vivo, results in activation of macrophages by allergen cytokine interleukin (IL)-12 and tumor necrosis factor-beta (TNF) [36,37]. Eugenol found in cinnamon and clove tends to bind protein covalently and acts as hapten. At higher concentrations, it has been found to be cytotoxic for fibroblast and osteoblast [38].

**Figure 3:**
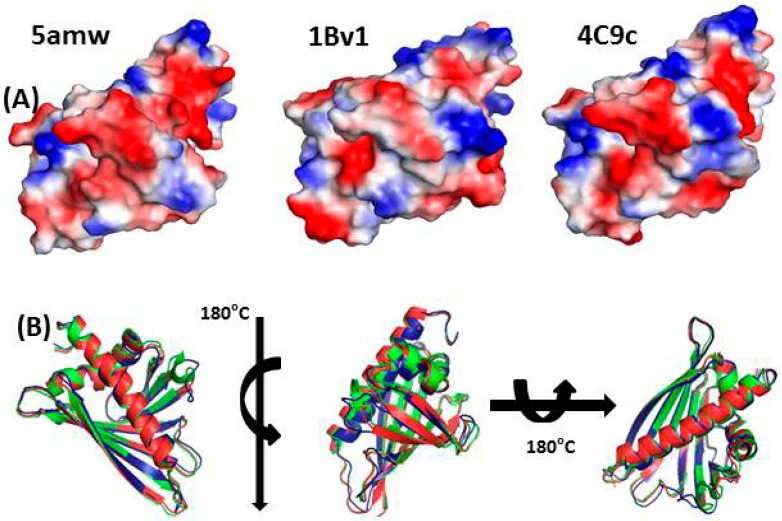
Structural conservation of the prolifin allergen from strawberry and birch (**A**): Pathogenesis-Related 10 (PR-10) Fra a 2 protein from Strawberry (PDB id- 5amw) [48]; Birch pollen Betv1protein(PDB id-1Bv1) [47]; Strawberry pathogenesis-related 10 (PR-10) Fra a 1E protein (PDB id-4C9c) [46] (**B**) Structural superposition of Fra a 2, Betv1 and Fra a 1E proteins; conservation in the overall structure elements is evident [Three dimensional structural files were retrieved from rcsb.org and images generated by PYMOL].

Generally, allergy to spice is prevalent after an initial sensitization with other allergens. An isolated case indicated occupational asthma through aniseed dust sensitization [39]. Cross-reactivity tends to occur due to the presence of common epitopes or similar immunogens, hence aggravating the clinical sensitivity. A distinct phylogenetic association has been observed as a major factor deciding the cross-reactive epitopes, especially in the case of protein and carbohydrate determinants (CCD). The validity of cross-reactivity became evident when a 27-year-old subject was diagnosed with an allergy to coconut, banana, and kiwi with allergic rhinitis to horse, cat, dog, and cow. Skin prick test, ELISA and bronchial inhalation challenges confirmed inhalation of dust from paprika, coriander, and mace induced asthma-like symptoms [40]. Bioinformatic analysis [structure comparison using PYMOL visualization tool] of some evident allergens in spice indicated overall conservation of epitopes (Figure 3) [41]. Carbohydrate moieties present on the proteins may induce cross-reactivity reactions and thus named as cross-reactive CCD [42,43]. Cross-reactivity between sesame seeds and poppy seeds was found to be very high as shared epitopes have been recognized [44] as has been an evident case of cashew and pistachio [42,43]. Additionally, from serum IgE analysis, cross-reactivity has been also observed in pink peppercorn (Brazilian peppers) and cashew nut which was mediated partly by 2S albumin seed storage proteins [45]. There are several types of proteinaceous allergens present in the spices like profilins, Betv1, CCD containing glycoproteins, germins, and pathogenesis-related proteins [43]. The Betv1 allergen family is structurally well defined and marked epitope conservation has been identified (Figure 3). The Betv1 analogs belong to PR-10 class of proteins and structurally resemble lipocalin-2 protein [46,47,48]. Lipocalins are transport proteins capable of carrying hydrophobic moieties across the membranes and hydrophobic molecule especially lipids are capable of inducing the innate immune response.

## 4. Molecular Assessment of Spice Allergy: How It All Starts?

A serendipitous identification, whereby spice-based allergy has found to be associated with occupational and domestic surroundings. According to the American College of Allergy, Asthma, and Immunology (ACAAI), 2% to 3% of the adult population and 8% in children younger than six years suffer from severe allergies to spices. Repeated confirmatory analysis has indicated females are more prone to allergic rhinitis than males (66.2% vs. 33.3%) [49]. Occupational skin diseases have been reported between 10% and 15% of all occupational diseases with significant economic impact [50]. Data from a voluntary Surveillance of Work-related and Occupational Respiratory Disease in South Africa (SORDSA) programme described 44 cases of occupational asthma (14.4%) reported in food handlers (October 1996 to June 2002). In a multi-centric study in India, 1097 out of 1860 patients were found to be allergic, with bronchial asthma prevalent in a range of 0.4%–4.8% [51].

### 4.1. Physiology of Spice Sensitization

Approximately 30% of the total airborne particulates are small enough to reach the distal airways [52]. Resuspension of settled dust is another dynamic process, and fluorescent biological aerosol particles (FBAPs) based studies have indicated an exposure level of dust ranging from 0.5 to 2 cm^−3^ (mass range: 50 to 600 μg/m^3^) [53]. The probability of small airborne particles (<500 nm) inhaled through nasal or oral passage depends on particle aerodynamic properties along with breathing mode and inhalation flow rate. Once inhaled, the deposited particles can be absorbed in blood by passive diffusion [54]. PM_2.5_ constitutes the inhalable particulate fraction and are especially dangerous as they penetrate through the body’s natural filters [55]. Earlier studies also reported that fraction of inhaled airborne particles could penetrate beyond the terminal bronchioles into the gas-exchange region of the lungs [56]. Woody spices upon grinding are cross-contaminated with free crystalline silica; cobalt and other metal dust and thus may initiate a mild case of systemic poisoning, although tachyphylaxis may not persist. Ultrafine (<100 nm) and large (>5000 nm) particles are dispersed and accumulate in the extrathoracic area and upper tracheobronchial regions. Bigger particles due to higher penetration depth tend to accumulate in the lung alveoli. Inhaled particles with aerodynamic diameter >30,000 nm are deposited in the nose via filtration through nasal hair and impaction during breathing. Depending on the dissolution parameter the class I and class II allergens may diffuse to the bodily fluids to elicit an antibody-mediated response. Inhalation of class II allergens increases the sensitization and activation of allergen-specific IgE releasing mast cells and macrophages. Pro-inflammatory mediators released by macrophages, in turn, increase the contraction of the airway smooth muscles along with the release of mucous. Hotter spices like chili and paprika are more likely to act as adjuvants for sensitization by promoting transport of molecules below a molecular mass of 70 kDa [57].

Some of the potential manifestations caused due to exposure to spice dust are rashes, and coughing has been observed after immediate exposure to airborne oregano, thyme, coriander, caraway seed, and cumin. A distinct cross-reactivity has been observed with food allergens and aeroallergens [58,59,60]. Cross-reactivity for protein allergens seems to be a major trigger for the stimulation of allergic reactions. Allergic sensitization to the Apiaceae family is commonly reported and known as ‘celery-mugwort spices’ syndrome [61]. In addition, sesquiterpene lactones, a secondary metabolite from the Asteraceae family cause an allergic response and presumably responsible for severe cross-sensitivity between tansy and chrysanthemum [26,62,63]. Celery-mugwort-spice syndrome along with mugwort-mustard-allergy syndrome has been found to be associated with weed pollinosis, which in turn has a wide spectrum of overlap [26,64].

The proteases digest most protein allergens; hence, IgE sensitization is only possible as an aeroallergen (through inhalation) of cross-reacting pollens, particularly mugwort and birch. Both linear and three-dimensional epitopes can induce an allergic reaction in organisms. Three-dimensional epitopes tend to sustain their structure upon inhalation and thus are capable of aggravating hypersensitive reactions in the organism [65]. Grinding and drying of the spices denature class 2 allergens, hence its associated allergenicity. However, a pathogenesis-related protein in paprika, osmotin (*P*-23) resists processing. A similar trend was observed in “celery-mugwort spices” syndrome with the members of anise, fennel, cumin, and coriander and Bet v1 and profilin homologs in poppy, even after roasting.

### 4.2. Sensors and Mediators of Spice Sensitization

Respiratory allergic reactions seem to be mediated by a family of proteins known as transient receptor potential (TRP) superfamily [66]. They have been classified into 28 different members in the mammals and act as polymodal sensors and ion channels [67]. In the presence of suitable stimuli, a cellular calcium influx is generated resulting in depolarization thus trigger voltage-dependent cellular processes [68]. TRP agonists tend to bind to the ankyrin motifs present of the extracellular surface of the protein and induce the activation of gated calcium channels [69]. TRPA1 agonists include extracts from spicy foods, such as mustard oil, garlic, cinnamon, black pepper, and wasabi. These TRP proteins are considered as the connection between the respiratory immune system and inflammation-inducing neuron system [70]. TRPA1 activation plays a crucial role in the mechanism for evoking cough associated with environmental and occupational exposure to respiratory irritants. Challenged with an antigen followed by activation of airway sensory nerves, triggers a central neuronal reflex is triggered leading to a parasympathetic cholinergic constrictor response. The data showed that blockade of the TRPA1, but not TRPV1, channels inhibited the LAR [71].

**Figure 4:**
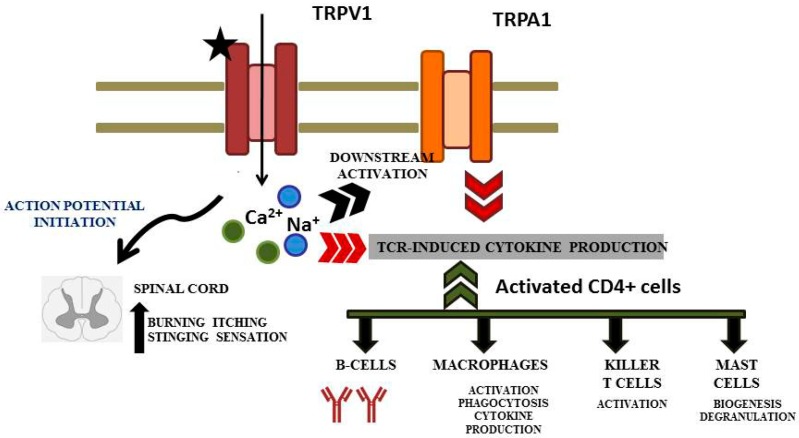
Overview of immune response elicited in response to binding of allergen to transient receptor potential TRPV receptors. Binding of agonist [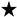] induces the activation and subsequent aggravation of the immune response.

TRPV1 channel agonists have been shown to cause bronchoconstriction in humans and animals; the response is thought to involve local ‘neurogenic inflammation’ and activation of a central reflex [71]. Sensory C fibers expressing TRPV1 were reported to show high co-localization between the neurokinins calcitonin gene-related peptide and substance P [72]. TRPV1 channels have a propensity to bind to different noxious metabolites is the region demarcated by TM2 through TM4 transmembrane domains addressed as the vanilloid binding pocket [73,74,75]. Capsaicin has been extensively studied and found to induce outward movements of S4-S5 linker between TM2 and TM3, thus activating it [76]. TRPV1, once activated by vanilloid molecules allows the influx of cations, as Ca^2+^ and Na^+^. TRPV1 induces apoptosis by activating calcium-dependent proteases, lipases, and nucleases. Simultaneously, it activates the TCR mediated immune response pathway resulting in activation and localization of B cells, killer T cells, macrophages and mast cells [77,78]. Proteinaceous allergens like BetV1 tends to bind ankyrin repeat of TRPA1, thus inducing the IL3 producing Th2 cells [79] (Figure 4).

## 5. Control Strategies and Assessment

As of now for spice allergens, no mandates for exposure limits and threshold limit values have been implemented globally. The USA has categorized spice dust particles as particulates not otherwise regulated (PNOR). In California, the OSHA permissible exposure limit (PEL) for PNOR is 10 mg/m^3^ for total (inhalable) dust [80]. An increase in nasal based symptoms has been found to be correlated to 1 mg/m^3^ whereas the risk of asthma was found to be at concentrations more than 3 mg/m^3^ concentration level (Wiley, 1997). Similarly, in Poland, PEL for organic dust are 2 mg/m^3^ for thoracic and 1 mg/m^3^ for respirable fractions in the presence of silica dispersants and its absence, acceptable values are 4 mg/m^3^ for thoracic and 2 mg/m^3^ for respirable fractions [81]. However, infused adulterants and pre-sensitization to an allergen tend to lower the requisite levels of PNORs further and hence have a tendency to aggravate allergic reaction in short time duration [82,83].

As occupational allergic outbreaks among exposed workers have intensified over the years, certain standards have been postulated by the government. Ministry of Environment and Forests under Environment (Protection) Rules and Emission Control System have limited particulate emission standards to 150 mg/nm^3^ with the height of 11 m above ground level and 2 m above roof level. Similarly, National ambient air quality standards (NAAQS) (Table 1) have set exposure assessment mechanisms of particulate matter.

Currently, testing and validation of the plausible allergens depend on double-blind placebo-based field trials; but the environmental conditions may lead to inconsistency of results. Hence, the trend is progressing towards the usage of allergen exposure unit (AEU) like Allergen Biocube [84]. AEC enables exposure of specific allergens in a controlled environment and tends to complement the field trials for allergen studies. AECs have been set up in the USA, Canada, Japan, Germany, France, Denmark, Poland, and Austria. Some specific standards have been set by the task force to maintain a uniformity of the conditions used in these AECs primarily being, duration of exposure, the frequency of challenges, level of allergen exposure, types of placebo and patients screened and their safety issues [85].

With the escalating levels of allergic manifestations, workplace control of exposure needs an integrated approach for control of PNOR emissions. Based on the individual survey and the meta-analysis on occupational exposure to airborne particulates in terms of spices, several controls have been formulated for the sake of implementation. Strategy for controlling measures have been designed on the ‘STOP principle’ which combines four components as systemic (S), technical (T), organizational (O) and personal (P) measures [86,87]. Systemic measures embrace control of procedural risk whereas technical measures involve minimization of the emission of spice particles in processing and seasoning. Organizational measures cover isolation of workstations, restriction of movement and operational sites to avoid high contamination levels, training to promote safe working habits, medical surveillance while personal measures may be kept on respiratory protection, personal protective equipment.

## 6. Conclusions

Spices have aromatic properties and are thus used as seasoning and food processing worldwide. Although beneficial for human health due to its enzyme stimulating characteristics spice particles emitted during various processes are a matter of concern as these suspended particulate matters are inhaled and settled particles come in contact with skin causes respiratory and allergic ailment. These ailments may be occupational asthma due to short term exposure and could lead to occupational dermatitis and rhinitis if failing to cure. In the same way, rendering skin contact allergy can occur depending on the long term and short-term exposure of spice dust. Spice dust has been categorized based on various definitions (Table 1) and concluded that spice dust might also be called as combustible dust due to its explosive nature.

However, there is no provision to reference and regulate the combustible dust directly in the national Health and Safety Executive legislation. Though it may be regulated indirectly concerning general clauses related to providing a safe workplace for workers, for instance, ventilation requirements, explosion suppression systems, etc. Thus, this is essential to understand the potential for combustible dust to manage the issue properly. So far, there is no comprehensive study on spices which can give a direction to policymakers to formulate the guidelines to prevent occupational health hazards. In this study, we have tried to investigate a collective approach in terms of spice, like the source of origin and size of suspended particulate matter; type of combustible dust and; the health effects due to airborne particles.

Future work will attempt to evaluate exposure-response relationships as it is imperative to characterize the occupational environment and its health effects.

## 7. Limitations of the Study

Time and financial constraints are the main limitations on the validity of the study, which require various intercessions such as engineering controls of dust emission, other industries of a similar nature, drawing up the questionnaire based on participant’s habits. The results of this study are corresponding to secondary sampling concentration levels and not to personal sampling concentrations as per potential participants.

## Figures and Tables

**Table 1:** National ambient air quality standards (NAAQS) and methods Source: http://cpcb.nic.in/air-quality-standard/.

Pollutants	Time Weighted Average	Concentration in Ambient Air		Methods of Measurement
		Industrial, Residential, Rural & other Areas	Ecologically Sensitive Area (Notified by Central Government)	
Particulate Matter (Size < 10 µm) or PM_10_ µg/m^3^	**Annual**	60	60	1. Gravimetric 2. TEOM 3. Beta attenuation
	24 H	100	100	
Particulate Matter (Size < 2.5 µm) or PM_2.5_ µg/m^3^	Annual	40	40	1. Gravimetric 2. TEOM 3. Beta attenuation
	24 H	60	60	
